# Trajectories of Children's Executive Function After Traumatic Brain Injury

**DOI:** 10.1001/jamanetworkopen.2021.2624

**Published:** 2021-03-19

**Authors:** Heather T. Keenan, Amy E. Clark, Richard Holubkov, Charles S. Cox, Linda Ewing-Cobbs

**Affiliations:** 1 Division of Critical Care, Department of Pediatrics, University of Utah, Salt Lake City; 2 Department of Pediatric Surgery, McGovern Medical School at Houston, The University of Texas Health Science Center at Houston, Houston; 3 Department of Pediatrics and Children’s Learning Institute, McGovern Medical School, The University of Texas Health Science Center at Houston, Houston

## Abstract

**Importance:**

Executive functions are critical for school and social success. Although these functions are adversely affected by pediatric traumatic brain injury (TBI), recovery patterns are not well established.

**Objective:**

To examine 3-year trajectories of selected children’s executive functions after TBI.

**Design, Setting, and Participants:**

This prospective cohort study was conducted from January 22, 2013, to September 30, 2015, with 3-year follow-up at the level I pediatric trauma centers Primary Children’s Hospital in Salt Lake City, Utah and Children’s Memorial Hermann Hospital in Houston, Texas. Study participants included children aged 2 to 15 years with TBI or orthopedic injury (OI) who were treated at the participating hospitals. Children were consecutively recruited and stratified by injury severity and age group. A total of 625 children consented and completed a baseline survey; 559 (89%) children completed at least 1 follow-up and composed the analysis cohort. It was hypothesized that recovery would differ by injury severity, age at injury, and sex. Data analyses were performed from June to October 2019.

**Main Outcomes and Measures:**

Growth curve models examined the pattern of change in the Emotional Control, Inhibit, Working Memory, and Plan-Organize subscales of the Behavior Rating Inventory of Executive Function (BRIEF) or BRIEF-Preschool. For all BRIEF subscales, higher scores indicate worse symptoms, and a score of 65 or greater represents clinical impairment.

**Results:**

A total of 559 children (mean [SD] age, 8.6 [4.4] years; 356 boys [64%], 328 non-Hispanic White children [59%]) were included in the study: 155 (28%) children had mild TBI, 162 (29%) had complicated mild or moderate TBI, 90 (16%) had severe TBI, and 152 (27%) had OI. Growth curve trajectories varied by BRIEF subscale and injury severity. Overall, children with TBI did not return to their preinjury baseline, with a stepwise worsening of each outcome at 36 months by TBI severity compared with OI. Among children with severe TBI, trajectories accelerated fastest, indicating increased problems, from injury to 12 months for the Emotional Control (9.0 points; 95% CI, 6.0-11.9 points), Inhibit (3.6 points; 95% CI, 1.6-5.6 points), and Working Memory (7.0 points; 95% CI, 4.1-9.9 points) subscales. Their trajectories plateaued, with a secondary acceleration before 36 months for the Emotional Control and Working Memory subscales. Children with mild TBI had worse 36-month scores on all subscales except Inhibit compared with OI. Recovery patterns were similar for boys and girls.

**Conclusions and Relevance:**

In this longitudinal cohort study of children with TBI, trajectory analysis revealed that some children worsen after a recovery plateau, suggesting a need for longitudinal reassessment beyond 1 year postinjury.

## Key Points

**Question:**

What is the 3-year trajectory of recovery of executive function after mild-to-severe traumatic brain injury in children?

**Findings:**

In this longitudinal cohort study of 559 children with mild, moderate, or severe traumatic brain injury or orthopedic injury, patterns of recovery differed by injury severity, age at injury, and executive function assessed. Overall, growth curve models showed children’s functioning worsening most sharply from baseline to 12 months; children with severe traumatic brain injury showed a secondary worsening between 24 and 36 months on some subscales.

**Meaning:**

Results suggest that changes in the recovery of children’s executive function over time will require repeated assessments in order to tailor interventions.

## Teaser

This cohort study uses data from pediatric trauma centers to assess the recovery of children’s executive function after experiencing mild-to-severe traumatic brain injury.

## Introduction

Traumatic brain injury (TBI) can adversely affect executive functions (EFs) that play a central role in both academic performance and social interactions.^1,2,3,4^ Executive functions are self-regulation skills that facilitate sustaining attention, resisting distraction, managing frustration, assessing the consequences of actions, and planning for the future.^5^ Executive functions develop by using neural networks traversing frontal-striatal circuits,^6^ which are frequently disrupted by TBI.^7,8^ Executive function development extends in a nonlinear fashion from infancy into young adulthood,^9^ with EF components having different developmental trajectories. Inhibition and behavior regulation accelerate rapidly during preschool years and continue to develop through adolescence.^5,10,11^ Metacognitive skills, such as working memory, increase gradually,^9^ whereas planning accelerates during late childhood and adolescence.^12^ Because skills in a rapid stage of development may be more vulnerable to disruption by TBI than more well-established skills,^13,14^ TBI sustained during periods of accelerated EF growth may be associated with greater deficit. Understanding how TBI influences the developmental trajectory of EF in children injured in different developmental periods is critically important to allow targeted intervention for behavior regulation and metacognitive skills.^15^

Executive functions are commonly assessed using performance-based measures and behavioral measures of underlying EF. The Behavior Rating Inventory of EF (BRIEF)^16^ is a parent-reported behavioral measure widely used to provide an ecological assessment of behavior regulation and metacognitive EF in everyday settings and may be particularly sensitive to posttraumatic difficulties.^17^ Prospective studies using EF ratings over the first 2 years after TBI consistently show a dose-dependent response: children with severe TBI (sTBI) have greater executive dysfunction than those with mild TBI (mTBI).^18,19^ Time course and extent of EF recovery are not established, with reports at 10 years postinjury showing mixed results.^20,21^ Some studies suggest no recovery of 1 or more EF components across the first 2 years after TBI,^18,19,22^ whereas others found gains.^23^ Persistent decrements in EF 5 to 7 years postinjury in children with sTBI suggest that even when recovery occurs, children’s EFs do not return to their preinjury trajectory.^24,25^

To our knowledge, most prior studies have not followed children longitudinally beyond 2 years after TBI, limiting our knowledge of recovery patterns. The current study fills this gap by modeling injury and demographic factors influencing children’s EF growth curves from baseline performance through 3 years postinjury, while accounting for preinjury abilities and family environment.^18,19,26^ We hypothesized (1) a dose-response relationship with TBI severity, by which children with severe injury would show greater initial decrement, improvement across the first year, and then deceleration; (2) that sex and age at injury would moderate the effect of TBI, with girls having greater difficulties over time and greater disruption of abilities rapidly developing at the time of injury; and (3) that positive family function and social capital would provide a protective recovery effect.

## Methods

This longitudinal, prospective cohort study conforms to the Strengthening the Reporting of Observational Studies in Epidemiology (STROBE) reporting guideline; however, the institutional review board prohibited recording information about families declining participation. The patient population included children with TBI or orthopedic injury (OI) without TBI who were recruited from the emergency department or inpatient setting at 2 level I pediatric trauma centers: Primary Children’s Hospital in Salt Lake City, Utah, and The University of Texas Health Science Center at Houston/Children’s Memorial Hermann Hospital. This study was conducted from January 22, 2013, through September 30, 2015, sequentially to fill strata of injury type, severity, and age group (0-30 months, 31 months-5 years, 6-11 years, and 12-15 years). Children aged 2 to 15 years who were eligible for the Behavior Rating Inventory of Executive Function (BRIEF) or BRIEF-Preschool (BRIEF-P) at the time of injury were included in this analysis. Children with severe developmental delay or psychiatric diagnoses requiring a closed classroom setting were excluded. Institutional review board approval was obtained from the University of Utah and The University of Texas Health Science Center at Houston. Parents and children provided written consent and assent.

### Definitions

Traumatic brain injury severity was measured using the lowest presenting emergency department pediatric Glasgow Coma Scale (GCS) score.^27^ Traumatic brain injury was categorized by severity: mTBI was defined as a GCS score greater than or equal to 13 upon presentation to health care, with a GCS score of 15 at discharge or after 24 hours if hospitalized; 1 or more focal signs including a period of transient confusion, loss of consciousness for 30 minutes or less, and/or transient neurological abnormalities.^28,29^ Mild TBI was subclassified as complicated mild based on computed tomography evidence of intracranial hemorrhage. Moderate TBI and sTBI were categorized by a GCS score of 9 to 12 and 3 to 8, respectively. Intubated and sedated children were scored 3T.

The comparison group included children with an upper or lower extremity long bone fracture without TBI who were recruited contemporaneously with the TBI group. Orthopedic injury comparisons isolated the outcomes of TBI from those of the injury alone. Trauma registrars assigned the Abbreviated Injury Scale.^30^

Parents completed surveys in English or Spanish of family demographic information, family functioning, social support, and child outcome measures a median of 8 days (interquartile range, 3-15 days) after injury to represent preinjury values. Follow-up assessments were collected at 3, 12, 24, and 36 months in person, online, or by telephone. Trained study coordinators abstracted medical records for clinical and injury mechanism data using standardized forms.

### Outcome Measures

The BRIEF or BRIEF-P was administered for children aged 5 to 15 years or younger than 5 years, respectively. The BRIEF has high test-retest reliability (0.82-0.88).^16,31^ The BRIEF and BRIEF-P behavior regulation (Inhibit, Emotional Control) and metacognitive (Working Memory, Plan-Organize) subscales were used because they are assessed in both BRIEF versions. Scores were combined across age versions to allow inclusion of children aging from BRIEF-P to BRIEF. BRIEF T-scores are based on normative data for age and sex (mean [SD] score, 50 [10]). The 90% CI range is 5 to 6 points. Scores greater than or equal to 65 represent clinical impairment. A change of 0.5 SD after TBI was considered clinically important.^19^

Family function was assessed using the McMaster Family Assessment Device–General Functioning Scale; scores range from 1 to 4, with higher scores represent worse functioning.^32^ The Social Capital Index total score measures a person’s perceptions of personal, family, neighborhood, and spiritual community support; scores range from 1 to 5, with higher scores representing more support.^33^ Families self-reported income, child race/ethnicity, and language preference. Income relative to US federal poverty level was calculated by family size.^34^

### Data Analysis

We compared patterns of change on the BRIEF and BRIEF-P subscales from baseline assessment through 36 months for children with TBI vs OI using growth curve models. Analyses were performed from June to October 2019. In these models, the intercept represents the level of the outcome at 12 months, whereas slope and curvature parameters represent the rate of change and the acceleration at that point. Time was centered at 1 year postinjury to facilitate interpretability, because the largest recovery gains generally occur within 1 year. All other continuous variables were centered at observed means. Complicated mild and moderate TBI were combined (cmmTBI) for modeling because of similarity of outcomes in other studies.^35^ Models fit included all available observations.

Trajectories were evaluated separately for each outcome. Initial evaluations focused on determining optimal covariance structure, assessing model fit using Akaike information criteria. Models including only fixed effects performed better than those fitting time using random effects. Final growth curve models were implemented in SAS, version 9.4 (SAS Institute Inc) specifying an unstructured covariance matrix across time points (preinjury, 3, 12, 24, and 36 months). Covariance matrix elements were estimated separately for children with TBI and OI; BRIEF Emotional Control showed additional fit improvement by further separating out sTBI in covariance modeling.

All models included the following independent variables a priori, regardless of significance: time since injury, injury type or severity, age at injury, child sex, preferred language, family function, and social capital. We evaluated interactions of sex × age, sex × injury group, and age × injury group. Finally, interactions with time up to a cubic effect were considered for all variables. During model development, interactions and site effect were trimmed from candidate models by iteratively eliminating those with *P* > .05 for inclusion. Candidate models including a given interaction retained all corresponding lower-order terms. For each outcome, the final reported model is the first model achieved with all remaining independent variables, excepting the a priori factors mentioned previously, having *P* < .05 for inclusion. All *P* values were 2-sided.

Results were summarized as mean contrasts over time and between groups with corresponding 95% CIs. We summarized growth curve patterns over time graphically by age, sex, and injury type or severity, assuming mean values for all other variables. Derivative graphs summarize rate of change over time. Because preferred language was associated with meaningful trajectories for some outcomes, Spanish language groups are presented separately in eFigures 2-5 in the Supplement.

## Results

The families of 625 children consented to and completed a baseline survey. A total of 559 (89%) children (mean [SD] age, 8.6 [4.4] years; 356 boys [64%], 328 non-Hispanic White children [59%]) completed at least 1 BRIEF or BRIEF-P follow-up and were included in the study (Table). One hundred fifty-five children (28%) had mTBI, 162 (29%) had cmmTBI, 90 (16%) had sTBI, and 152 (27%) had OI (eFigure 1 in the Supplement). Assessments were completed at 3 months (n = 517), 12 months (n = 511), 24 months (n = 473), and 36 months (n = 416). Children completing follow-up were more likely to be from Utah (325 [85%] vs 234 [42%] from Texas), of White race/ethnicity (328 [59%] vs 151 [27%] Hispanic children, 40 [7%] Black children, and 34 [6%] other), and have better social capital (mean [SD] Social Capital Index score, 3.5 [1.0] vs 3.0 [1.1]) and family functioning (mean [SD] McMaster subscale score, 1.5 [0.5] vs 1.7 [0.5]) than those who did not (eTable 1 in the Supplement). Pearson correlation coefficients across EF domains ranged from 0.58 to 0.82.

**Table.** Description of Cohort by Injury Group^a^

Characteristic	No. (%)^b^				
	Mild TBI (n = 155)	Complicated mild/moderate (n = 162)	Severe TBI (n = 90)	Orthopedic (n = 152)	Overall (N = 559)
Enrollment site: Texas	62 (40)	66 (41)	37 (41)	69 (45)	234 (42)
Age at injury, y					
2-5	61 (39)	57 (35)	30 (33)	62 (41)	210 (38)
6-11	49 (32)	55 (34)	32 (36)	46 (30)	182 (33)
12-15	45 (29)	50 (31)	28 (31)	44 (29)	167 (30)
Girls	59 (38)	62 (38)	24 (27)	58 (38)	203 (36)
Boys	96 (62)	100 (62)	66 (73)	94 (62)	356 (64)
Preferred language, Spanish	20 (13)	10 (6)	14 (16)	24 (16)	68 (12)
Child race/ethnicity					
Hispanic or Latino	38 (25)	34 (21)	30 (34)	49 (32)	151 (27)
Non-Hispanic White	91 (59)	105 (66)	50 (57)	82 (54)	328 (59)
Non-Hispanic Black	14 (9)	9 (6)	6 (7)	11 (7)	40 (7)
Non-Hispanic other^c^	11 (7)	12 (8)	2 (2)	9 (6)	34 (6)
Income relative to poverty level, mean (SD)	2.8 (2.1)	3.1 (1.9)	2.4 (1.6)	2.7 (2.0)	2.8 (1.9)
McMaster family functioning, mean (SD) score^d^	1.5 (0.5)	1.6 (0.4)	1.5 (0.4)	1.5 (0.5)	1.5 (0.5)
Social Capital Index, mean (SD) score^e^	3.5 (1.1)	3.5 (1.0)	3.4 (1.0)	3.5 (1.1)	3.5 (1.0)
Preexisting attention or learning problems	19 (12)	19 (12)	13 (14)	11 (7)	62 (11)
Injury mechanism					
Assault	0	2 (1)	0	0	2 (0)
Pedestrian or bicycle	23 (15)	19 (12)	16 (18)	7 (5)	65 (12)
Motorized vehicle	37 (24)	41 (25)	53 (59)	10 (7)	141 (25)
Fall	70 (45)	79 (49)	11 (12)	114 (75)	274 (49)
Struck by or against	13 (8)	12 (7)	5 (6)	8 (5)	38 (7)
Organized sport	10 (6)	4 (2)	2 (2)	11 (7)	27 (5)
Other	2 (1)	5 (3)	3 (3)	2 (1)	12 (2)
Lowest ED GCS after resuscitation, median (IQR)	15 (15-15)	15 (14-15)	3 (3-6)	NA	15 (10-15)
Head and neck AIS, median (IQR)	1 (1-2)	3 (3-3)	4 (3-5)	0 (0-0)	2 (0-3)
Other area injured aside from head	76 (49)	123 (76)	82 (91)	152 (100)	433 (77)
Max AIS excluding head, median (IQR)	0 (0-2)	1 (1-1)	2 (1-3)	2 (2-3)	2 (1-2)

Abbreviations: AIS, Abbreviated Injury Scale; ED, emergency department; GCS, Glasgow Coma Scale; IQR, interquartile range; Max, maximum; NA, not available; TBI, traumatic brain injury.

^a^ Missing values were excluded from summary statistics as follows: child race/ethnicity (n = 6), income relative to poverty level (n = 39), Social Capital Index (n = 14), preexisting attention or learning problems (n = 1).

^b^ Values are expressed as No. (%) unless otherwise specified.

^c^ Non-Hispanic other includes African American, American Indian, Pacific Islander, Asian, and those reporting mixed race.

^d^ Using the McMaster Family Assessment Device–General Functioning Scale; scores range from 1 to 4, with higher scores representing worse functioning.

^e^ Social Capital Index scores range from 1 to 5, with higher scores representing more support.

**Figure 1.**
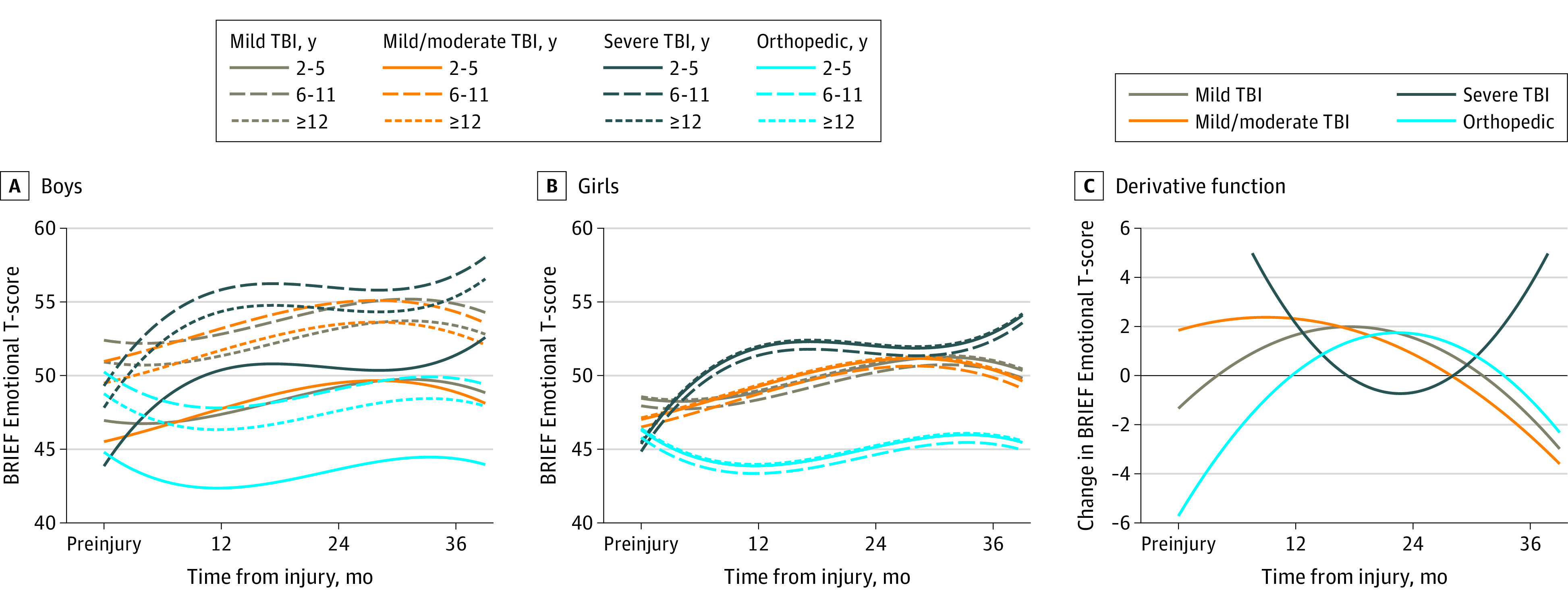
Emotional Control Outcomes Over Time by Injury Group and Age Estimated mean outcomes for (A) boys and (B) girls of English-speaking families assuming mean values of family function and social capital. Panel C shows the change in outcome over time. Values above 0 indicate that the score is increasing (worsening), whereas values below 0 indicated the score is decreasing (improving). BRIEF indicates Behavior Rating Inventory of Executive Function; TBI, traumatic brain injury.

**Figure 2.**
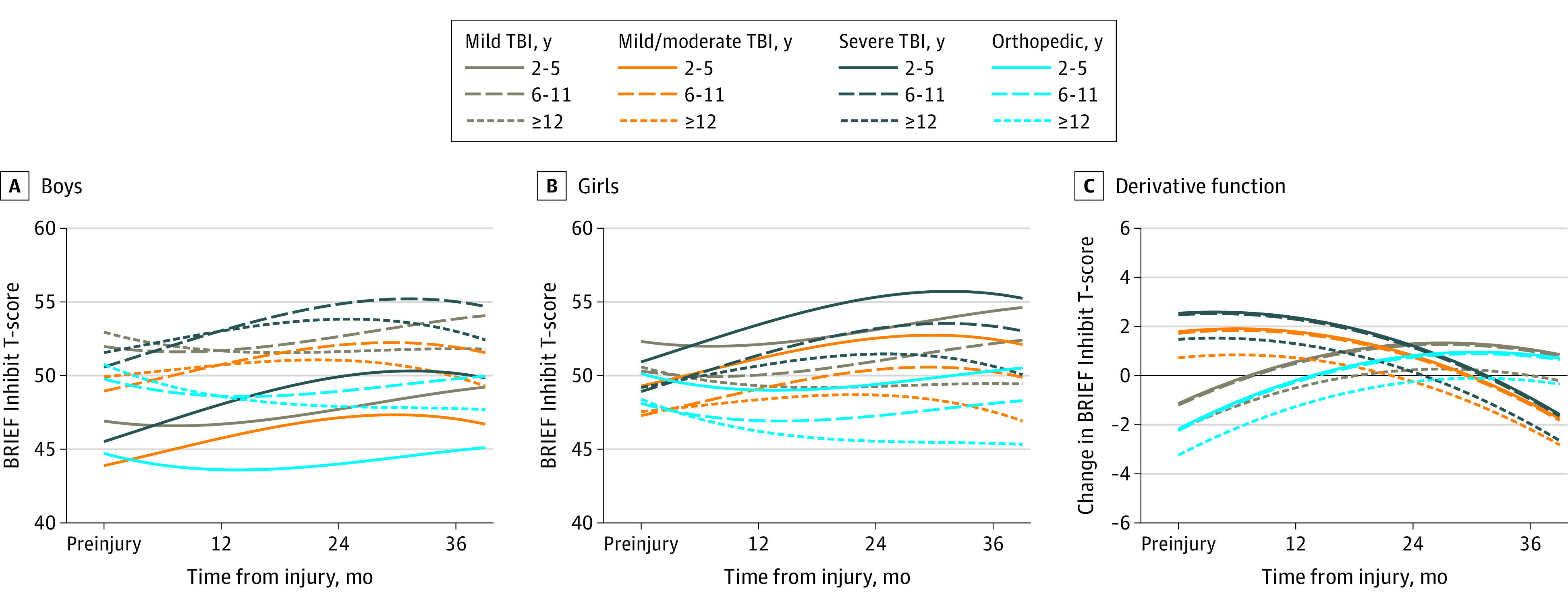
Inhibit Outcomes Over Time by Injury Group and Age Estimated mean outcomes for (A) boys and (B) girls of English-speaking families assuming mean values of family function and social capital. Panel C shows the change in outcome over time. Values above 0 indicate that the score is increasing (worsening), whereas values below 0 indicated the score is decreasing (improving). BRIEF indicates Behavior Rating Inventory of Executive Function; TBI, traumatic brain injury.

**Figure 3.**
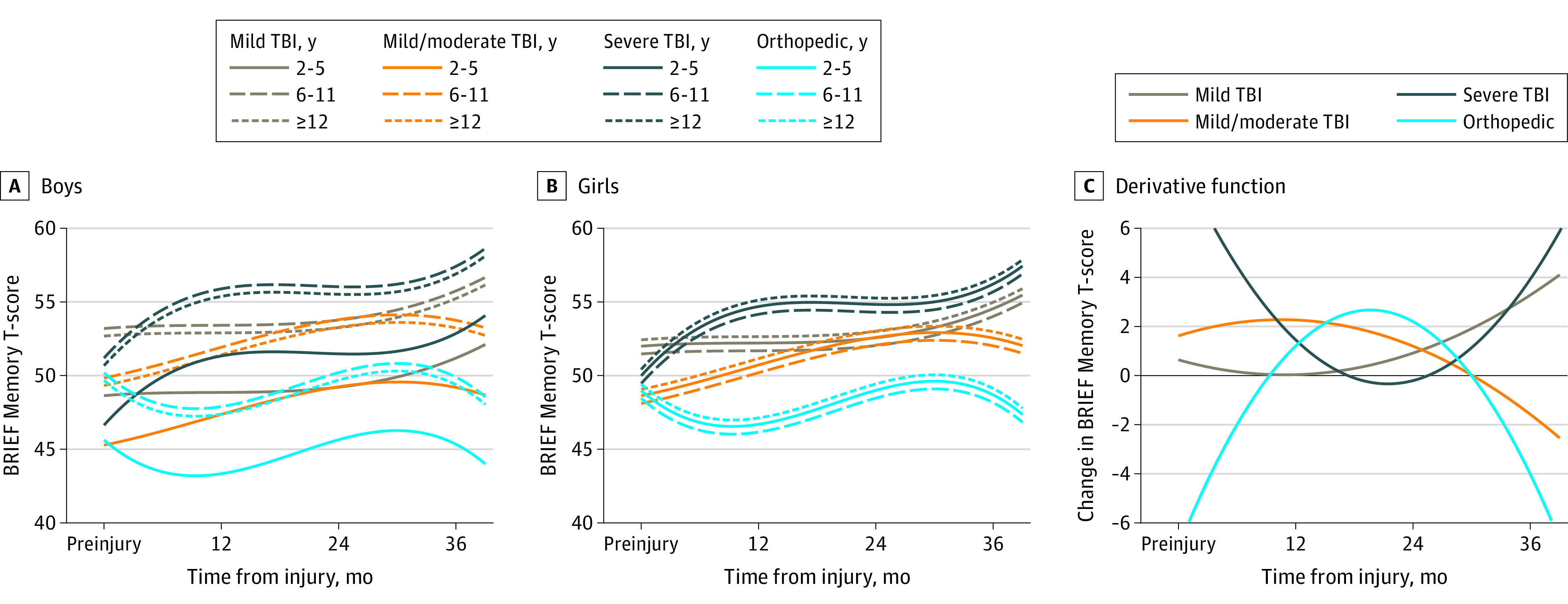
Working Memory Outcomes Over Time by Injury Group and Age Estimated mean outcomes for (A) boys and (B) girls of English-speaking families assuming mean values of family function and social capital. Panel C shows the change in outcome over time. Values above 0 indicate that the score is increasing (worsening), whereas values below 0 indicated the score is decreasing (improving). BRIEF indicates Behavior Rating Inventory of Executive Function; TBI, traumatic brain injury.

**Figure 4.**
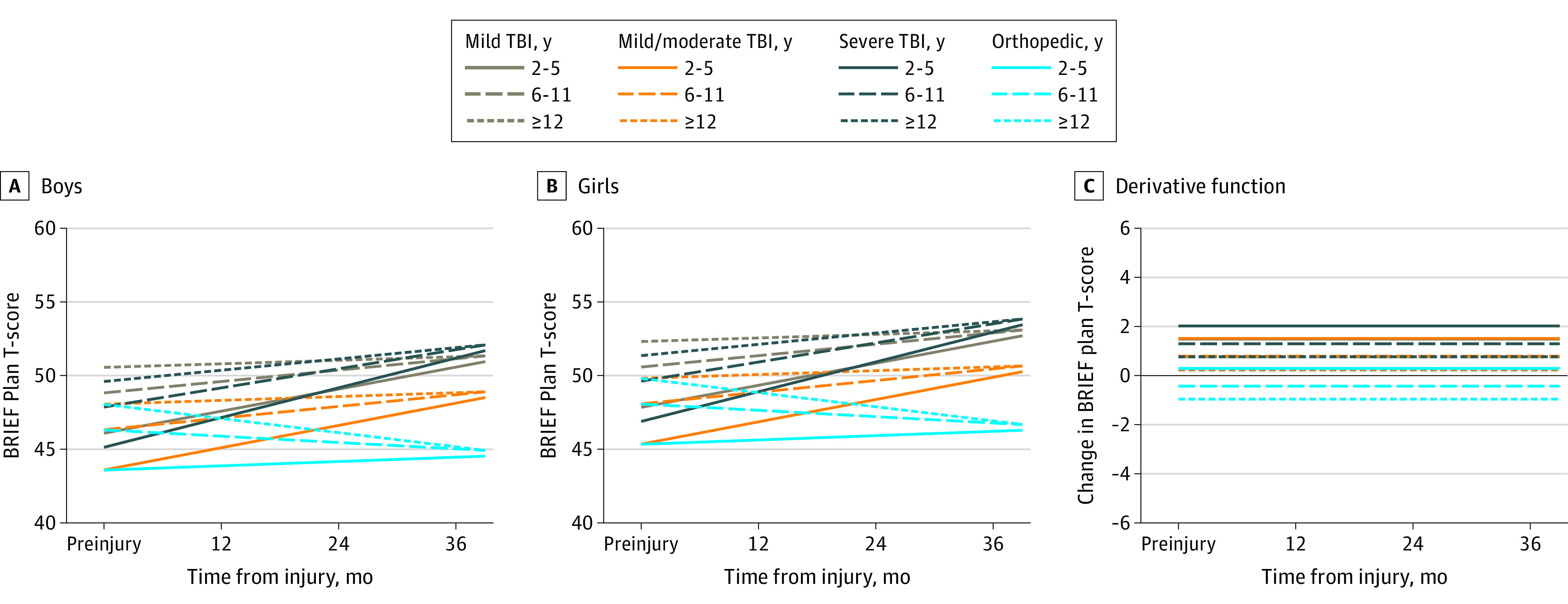
Plan-Organize Outcomes Over Time by Injury Group and Age Estimated mean outcomes for (A) boys and (B) girls of English-speaking families assuming mean values of family function and social capital. Panel C shows the change in outcome over time. Values above 0 indicate that the score is increasing (worsening), whereas values below 0 indicated the score is decreasing (improving). BRIEF indicates Behavior Rating Inventory of Executive Function; TBI, traumatic brain injury.

Model parameters, including interaction terms, are displayed in eTable 2 in the Supplement. Adjusted growth curves by sex, age at injury group, injury type, and TBI severity for each subscale are displayed in Figure 1, Figure 2, Figure 3, and Figure 4, with boys and girls presented separately. These figures show predicted scores over time for children of English-speaking families, with family function and social capital fixed at mean cohort values. Derivative functions of each growth curve, inclusive of both sexes, describe the rate of change for each outcome over time: if the line is above 0 (y-axis) at a particular postinjury time point (x-axis), the modeled outcome is increasing (worsening) at that time point from its current value; lines below 0 mean the outcome is decreasing (improving).

Growth curve shapes can be compared with the quantitative data in eTable 3 in the Supplement. For all subscales, poorer baseline family function was associated with consistently worse outcomes at 12 and 36 months postinjury, although this association appeared to attenuate over time. For every 1 point increase in family function, scores increased for the Inhibit T-score at 12 months (mean [SE] 5.95 [0.89]) and 36 months (4.18 [1.16]); Emotional T-score at 12 months (6.08 [0.91]) and 36 months (4.22 [1.26]); Working Memory T-score at 12 months (5.72 [0.92]) and 36 months (3.81 [1.31]); and Plan-Organize T-score at 12 months (5.48 [0.86]) and 36 months (4.04 [1.14]).

Higher social capital was associated with better outcomes at each time point. For each point increase in social capital, the BRIEF Inhibit T-score was decreased by 1.04 points (SE, 0.39) at each 12 and 36 months; Emotional T-score was decreased by 1.22 points (SE, 0.40) at 12 and 36 months, Working Memory T-score was decreased by 1.66 (SE, 0.50) at 12 months and 0.84 (SE, 0.58) at 36 months, and Plan-Organize T-score was decreased by 1.03 points (SE, 0.38) at 12 and 36 months (eTable 4 in the Supplement).

For the Emotional Control subscale, compared with children with OI, growth curves of children with sTBI accelerated over the first year (suggesting greater EF dysfunction), plateaued between 12 and 24 months, and then accelerated again by the 36-month time point (Figure 1). eTable 3 in the Supplement summarizes these differences in change over time, including the steep rise reflected in the 12-month change in scores from preinjury (9.0 mean points greater than OI; 95% CI, 6.0-11.9 points), the plateau between 12 and 24 months (−1.2 points; 95% CI, −3.7 to 1.4 points), and the slight rise from 24 to 36 months (0.2 points; 95% CI, −2.9 to 3.3 points). Compared with children with OI, children with sTBI had a mean 8.0-point increase (95% CI, 4.1-11.8) by 36 months. The derivative curve (Figure 1C) shows children with sTBI transitioning to improvement between 12 and 24 months, a flattening around 24 months (demonstrating the plateau seen in the growth curves), and finally returning above the 0 line and steepening, showing worsening during later follow-up. Children with mTBI and cmmTBI had slow acceleration of growth curves until around 24 months, but then improved. Children with mTBI (mean points, 2.9; 95% CI, 0.2-5.6) and cmmTBI (mean points, 3.8; 95% CI, 1.1-6.5) had a greater increase in scores from baseline relative to OI controls at 36 months; mTBI curves slowly accelerated until around 24 months, then improved. Group type did not interact with age or sex. The sex × age interaction was significant with boys aged 6 to 11 years; they had higher scores than girls or boys of other ages within the same injury group (Figure 1A).

For the Inhibit subscale, curves for children with mTBI paralleled those with OI in all age groups, with a gradual increase in scores over time (Figure 2) and similar changes from baseline to 36 months (mean points, 1.9; 95% CI, −0.5 to 4.2). Curves for children with cmmTBI and sTBI accelerated from injury to 12 months (mean points, 3.0 [95% CI, 1.3-4.7] and 3.6 [95% CI, 1.6-5.6], respectively). Scores then slowly accelerated from 24 to 36 months. Children with cmmTBI (mean points, 3.0; 95% CI, 0.6-5.3) and sTBI (mean points, 4.4; 95% CI, 1.6-7.3) performed worse at 36 months relative to baseline compared with the OI group. The age × time interaction indicates that children aged 2 to 5 years (mean points, 3.2; 95% CI, 1.0-5.4) and 6 to 11 years (mean points, 3.0; 95% CI, 0.7-5.3) performed worse than children aged 12 to 15 years during follow-up. The significant age at injury × sex interaction indicates lower scores among boys in the youngest age group compared with girls and older boys.

For the Working Memory subscale, scores of children with cmmTBI and sTBI accelerated in the first year postinjury, with sTBI having the steepest rate of rise (7.0 points; 95% CI, 4.1-9.9 points) (Figure 3). From 12 to 24 months, scores decelerated for both cmmTBI and sTBI groups; the mTBI and sTBI group then accelerated from 24 to 36 months. There was a stepwise increase in scores relative to baseline by severity for the TBI groups at 36 months compared with OI. The mean difference from preinjury to 36 months between OI and mTBI was 2.8 points (95% CI, 0.2-5.5); complicated mTBI, 4.2 points (95% CI, 1.5-6.9); and sTBI, 6.5 points (95% CI, 3.3-9.8) (eTable 3 in the Supplement). The age at injury by sex interaction is shown by the differing baseline scores; however, recovery patterns were similar by sex and age of injury.

For the Plan-Organize subscale, growth curve models were simpler than for the other outcomes. All time effects were linear, implying that predicted outcomes consistently increased or decreased over time in each injury and age subgroup. Flat derivative curves reflect this constant rate of change over time (Figure 4). Children in all TBI groups worsened. The age × time interaction indicates children aged 2 to 5 years had a steeper increase (worsening) in outcomes over time compared with children aged 12 to 15 years. At 36 months, children aged 2 to 5 years had a 3.8-point increase (95% CI, 1.4-6.1 points), whereas children aged 6 to 11 years were similar to the oldest group (mean point increase, 1.6; 95% CI, −0.8 to 4.0). Within injury and age groups, girls had higher scores than boys at baseline and all later time points, on average.

Growth and derivative function curves for families with a Spanish language preference are displayed in eFigures 2, 3, 4, and 5 in the Supplement. Overall, Spanish speakers started from a better baseline compared with English speakers. For the Emotional Control and Plan-Organize subscales, Spanish and English speakers had similar recovery patterns. Patterns differed for the Inhibit and Working Memory subscales. For Inhibit, OI and TBI groups improved but then worsened after 24 months in parallel (eFigure 3 in the Supplement). For Working Memory, derivative curves showed all TBI severities worsening over the first 12 months, then improving with some groups starting to worsen after 30 months (eFigure 4 in the Supplement).

## Discussion

Our study of EF growth curves during the first 3 years after TBI found differing patterns of children’s recovery depending on injury severity, age, and the EF subscale assessed. Consistent with prior literature, there was stepwise worsening of scores for children with mTBI, cmmTBI, and sTBI compared with controls with OI.^18,19,23,24^ Across outcomes, trajectories varied over time for TBI groups; however, children worsened most sharply from baseline to 12 months. Some subgroups, particularly sTBI, showed a secondary acceleration between 24 and 36 months. Children with all severities of TBI did not fully recover to their preinjury level of functioning.

Recovery patterns differed by EF subscale and injury group. After cmmTBI and sTBI, Inhibit scores worsened until 24 months and then plateaued. This contrasts with the Emotional Control and Working Memory subscales, which showed improvement after 24 months for children with cmmTBI but an increase in problems for sTBI between 24 and 36 months. Plan-Organize scores steadily worsened over the 3-year period. Children with mTBI had persisting parent-reported decrements 3 years after injury in all subscales except Inhibit. Because mTBI is common, even small persisting decrements are important at the population level.

We hypothesized that age at injury and sex would moderate the effect of TBI on EF outcomes. Age × time interactions indicated that Inhibit scores increased over time for children aged 2 to 11 years but decelerated for adolescents. This finding is consistent with prior studies that (1) reported greater inhibition deficits in infants and preschoolers than in older children^14^ and (2) noted relative sparing in late childhood compared with early childhood and adolescence.^17,22^ Plan-Organize scores were initially lower in children aged 2 to 5 years and accelerated across follow-up at a faster rate than in older children and adolescents. Adolescents were not selectively vulnerable to EF worsening. Our finding of vulnerability of specific EF in preschoolers that increased over time should be examined in relation to an uninjured comparison group.

Sex differences in the development of some EFs vary by age in cross-sectional studies^36^ but have not been described longitudinally. Age at injury interacted with sex for the Inhibit, Working Memory, and Emotional Control subscales.^17,19,22^ Scores differed by sex within age groups at baseline, and although these initial differences translated to outcomes at 36 months, patterns of recovery between boys and girls were similar. Future confirmatory studies of sex-specific recovery patterns with these and other outcomes are needed.

Children with good EF development are more likely to succeed in school, home, and social settings than those with EF difficulties.^37,38,39^ Similar to other studies, our BRIEF mean scores for children with TBI were within population norms. However, trajectories of children with TBI diverged from the OI group, indicating small to larger effect sizes, and did not return to their preinjury levels. Protective factors in this study included good family function and high social capital, suggesting that a family-centered approach promotes children’s long-term success. Across the TBI severity spectrum, children’s development of EF should be monitored; as some EFs may worsen again after a plateau, targeted assessment may identify the need for cognitive or socioemotional supports.

### Limitations

This study has some limitations. All assessments were by parent report, which measures reflecting behaviors associated with EF in everyday settings. However, ratings are subjective, which may lead to underreporting or overreporting in all groups. Ecologic and direct EF tests correlate poorly, making it difficult to know whether they measure the same construct, a recognized problem with EF measurement overall.^40^ Children participating in the follow-up had overall better family function, potentially biasing our study toward better outcomes. Differences in patterns between Spanish and English speakers on some subscales require further study.

### Conclusions

This cohort study assessed the recovery of children’s EF after experiencing mild-to-severe TBI. Study results suggest that children with all severities of TBI have EF decrements as long as 3 years after injury, with some experiencing a secondary increase in EF scores after an initial plateau. Results further suggest that children with TBI may struggle over time as tasks become more complex, leading to a need for reassessment and different supports to improve participation in the school, home, and community.

## Notes

Supplement. **eTable 1.** Description of Cohort by Follow Up Status **eTable 2.** BRIEF Outcome Models **eTable 3.** BRIEF Outcomes, TBI vs Orthopedic Injury Group, Preinjury to 36 Months **eTable 4.** Effect of Preinjury Family Characteristics on BRIEF Outcomes **eFigure 1.** Cohort Flow Diagram **eFigure 2.** BRIEF Emotional Control Outcomes Over Time by Injury Group and Age (Spanish Language Preference) **eFigure 3.** BRIEF Inhibit Outcomes Over Time by Injury Group and Age (Spanish Language Preference) **eFigure 4.** BRIEF Memory Working Outcomes Over Time by Injury Group and Age (Spanish Language Preference) **eFigure 5.** BRIEF Plan-Organize Outcomes Over Time by Injury Group and Age (Spanish Language Preference)
