# Supplementary material for: Trajectories of Children's Executive Function After Traumatic Brain Injury
Source: JAMA Netw Open. 2021 Mar 19;4(3):e212624. doi: 10.1001/jamanetworkopen.2021.2624 (PMC7980098; doi:10.1001/jamanetworkopen.2021.2624)
Supplement: Supplement. — eTable 1. Description of Cohort by Follow Up Status eTable 2. BRIEF Outcome Models eTable 3. BRIEF Outcomes, TBI vs Orthopedic Injury Group, Preinjury to 36 Months eTable 4. Effect of Preinjury Family Characteristics on BRIEF Outcomes eFigure 1. Cohort Flow Diagram eFigure 2. BRIEF Emotional Control Outcomes Over Time by Injury Group and Age (Spanish Language Preference) eFigure 3. BRIEF Inhibit Outcomes Over Time by Injury Group and Age (Spanish Language Preference) eFigure 4. BRIEF Memory Working Outcomes Over Time by Injury Group and Age (Spanish Language Preference) eFigure 5. BRIEF Plan-Organize Outcomes Over Time by Injury Group and Age (Spanish Language Preference) [file jamanetwopen-e212624-s001.pdf]

Supplementary Online Content

Keenan HT, Clark AE, Holubkov R, Cox CS Jr, Ewing-Cobbs L. Trajectories of children's executive function after traumatic brain injury. *JAMA Netw Open*. 2021;4(3):e212624. doi:10.1001/jamanetworkopen.2021.2624

eTable 1. Description of Cohort by Follow Up Status

eTable 2. BRIEF Outcome Models

eTable 3. BRIEF Outcomes, TBI vs Orthopedic Injury Group, Preinjury to 36 Months

eTable 4. Effect of Preinjury Family Characteristics on BRIEF Outcomes

eFigure 1. Cohort Flow Diagram

eFigure 2. BRIEF Emotional Control Outcomes Over Time by Injury Group and Age (Spanish Language Preference)

eFigure 3. BRIEF Inhibit Outcomes Over Time by Injury Group and Age (Spanish Language Preference)

eFigure 4. BRIEF Memory Working Outcomes Over Time by Injury Group and Age (Spanish Language Preference)

eFigure 5. BRIEF Plan-Organize Outcomes Over Time by Injury Group and Age (Spanish Language Preference)

This supplementary material has been provided by the authors to give readers additional information about their work.

© 2021 Keenan HT et al. *JAMA Network Open.*

eTable 1. Description of Cohort by Follow Up Status

	Follow up completed		
	**No (N = 66)**	**Yes (N = 559)**	**P-value[a]**
**Enrollment site**			<0.001
Utah	17 (26%)	325 (58%)	
Texas	49 (74%)	234 (42%)	
**Age (years) at injury**, mean (SD)	9.3 (4.9)	8.6 (4.4)	0.21
**Child sex**			0.09
Boy	49 (74%)	356 (64%)	
Girl	17 (26%)	203 (36%)	
**Preferred language**			0.99
English	58 (88%)	491 (88%)	
Spanish	8 (12%)	68 (12%)	
**Child race/ethnicity**			<0.001
Hispanic or Latino	24 (37%)	151 (27%)	
White, non-Hispanic	22 (34%)	328 (59%)	
Black, non-Hispanic	9 (14%)	40 (7%)	
Other, non-Hispanic	10 (15%)	34 (6%)	
**Income relative to poverty level**, mean (SD)	2.5 (2.0)	2.8 (1.9)	0.35
**McMaster family functioning**, mean (SD)	1.7 (0.5)	1.5 (0.5)	0.004
**Social capital index**, mean (SD)	3.0 (1.1)	3.5 (1.0)	<0.001
**Injury group and severity**			0.72
Mild TBI	19 (29%)	155 (28%)	
Complicated mild/moderate	17 (26%)	162 (29%)	
Severe TBI	14 (21%)	90 (16%)	
Orthopedic	16 (24%)	152 (27%)	

[a] P-value based on chi-square test for categorical variables and t-test for continuous variables.

© 2021 Keenan HT et al. *JAMA Network Open.*

eTable 2. BRIEF Outcome Models

**Outcome**	**Model terms included[a]**	**P-value[b]**
**Emotional Control**	Injury Group * $Time^3$	p=0.04
	Age at Injury * Child Sex	p=0.01
	Preferred language	p<0.001
	Family function * Time	p=0.01
	Social capital	p=0.003
**Inhibit**	Injury Group * $Time^2$	p=0.01
	Age at Injury * Child Sex	p<0.001
	Age at Injury * Time	p=0.01
	Preferred language * $Time^3$	p=0.03
	Family function * Time	p=0.01
	Social capital	p=0.01
**Working Memory**	Injury Group * $Time^3$	p=0.001
	Age at Injury * Child Sex	p=0.03
	Preferred language * $Time^3$	p=0.03
	Family function * Time	p=0.01
	Social capital * $Time^3$	p=0.004
**Plan /organize**	Injury Group * Time	p=0.001
	Age at Injury * Time	p=0.01
	Child Sex	p=0.03
	Preferred language	p=0.002
	Family function * Time	p=0.04
	Social capital	p=0.01

[a] All models included the a priori predictors: time since injury, injury type and severity, age at injury, child sex, preferred language, family function and social capital. Site was not important in any model. Model terms shown include only the highest interaction term in the model. All related lower-order terms are also included. For example, for BRIEF Inhibit, the higher order term Injury Group*$Time^2$ implies the inclusion of Time, $Time^2$, InjuryGroup, InjuryGroup*Time, and Injury Group*$Time^2$.

[b] P-value reflects overall significance of interaction term or main effect shown.

© 2021 Keenan HT et al. *JAMA Network Open.*

eTable 3. BRIEF Outcomes, TBI vs Orthopedic Injury Group, PreInjury to 36 Months

	Differences in change over time[a]			
**BRIEF T-score**	**Pre-injury to 12 months**	**12 to 24 months**	**24 to 36 months**	**Pre-injury to 36 months**
**Emotional Control**				
Mild vs Ortho	2.9 (0.4, 5.3)	0.6 (-1.3, 2.5)	-0.5 (-2.3, 1.3)	2.9 (0.2, 5.6)
cMild vs Ortho	4.7 (2.3, 7.1)	0.5 (-1.4, 2.4)	-1.4 (-3.2, 0.5)	3.8 (1.1, 6.5)
Severe vs Ortho	9.0 (6.0, 11.9)	-1.2 (-3.7, 1.4)	0.2 (-2.9, 3.3)	8.0 (4.1, 11.8)
**Inhibit**				
Mild vs Ortho	0.9 (-0.8, 2.6)	0.6 (-0.2, 1.4)	0.3 (-1.1, 1.8)	1.9 (-0.5, 4.2)
cMild vs Ortho	3.0 (1.3, 4.7)	1.0 (0.2, 1.8)	-1.0 (-2.5, 0.5)	3.0 (0.6, 5.3)
Severe vs Ortho	3.6 (1.6, 5.6)	1.5 (0.5, 2.4)	-0.7 (-2.6, 1.2)	4.4 (1.6, 7.3)
**Working Memory**				
Mild vs Ortho	2.5 (0.1, 4.9)	-2.0 (-3.9, 0.03)	2.3 (0.6, 4.0)	2.8 (0.2, 5.5)
cMild vs Ortho	4.4 (2.0, 6.8)	-0.4 (-2.4, 1.6)	0.3 (-1.5, 2.0)	4.2 (1.5, 6.9)
Severe vs Ortho	7.0 (4.1, 9.9)	-2.2 (-4.5, 0.2)	1.7 (-0.5, 3.9)	6.5 (3.3, 9.8)
**Plan/Organize**				
Mild vs Ortho	1.2 (0.4, 2.0)	1.2 (0.4, 2.0)	1.2 (0.4, 2.0)	3.6 (1.2, 6.0)
cMild vs Ortho	1.2 (0.4, 2.0)	1.2 (0.4, 2.0)	1.2 (0.4, 2.0)	3.7 (1.2, 6.1)
Severe vs Ortho	1.7 (0.7, 2.7)	1.7 (0.7, 2.7)	1.7 (0.7, 2.7)	5.2 (2.2, 8.2)

[a] The mean differences shown reflect a difference between groups for the difference over time. For example, for BRIEF Inhibit, the Severe group T-score increased (worsened) by 4.4 points more than the Orthopedic group from pre-injury to 36 months, on average.

© 2021 Keenan HT et al. *JAMA Network Open.*

eTable 4. Effect of Preinjury Family Characteristics on BRIEF Outcomes

	Pre-injury mean (SE)	12 months mean (SE)	36 months mean (SE)
**BRIEF Inhibit T-score**			
Social capital Index (1 point increase)	-1.04 (0.39)	-1.04 (0.39)	-1.04 (0.39)
Family function (1 point increase)	6.83 (0.92)	5.95 (0.89)	4.18 (1.16)
**BRIEF Emotional T-score**			
Social capital Index (1 point increase)	-1.22 (0.40)	-1.22 (0.40)	-1.22 (0.40)
Family function (1 point increase)	7.00 (0.94)	6.08 (0.91)	4.22 (1.26)
**BRIEF Working Memory T-score**			
Social capital Index (1 point increase)	-0.78 (0.43)	-1.66 (0.50)	-0.84 (0.58)
Family function (1 point increase)	6.67 (0.93)	5.72 (0.92)	3.81 (1.31)
**BRIEF Plan/Organize T-score**			
Social capital Index (1 point increase)	-1.03 (0.38)	-1.03 (0.38)	-1.03 (0.38)
Family function (1 point increase)	6.20 (0.91)	5.48 (0.86)	4.04 (1.14)

For all children, higher (worse) family functioning at pre-injury is consistently related to higher (worse) outcomes throughout the 3 years of follow up. This effect attentuates over time. Higher (better) social capital at pre-injury is consistently related to lower (better) outcomes throughout the 3 years of follow up.

© 2021 Keenan HT et al. *JAMA Network Open.*

eFigure 1. Cohort flow diagram

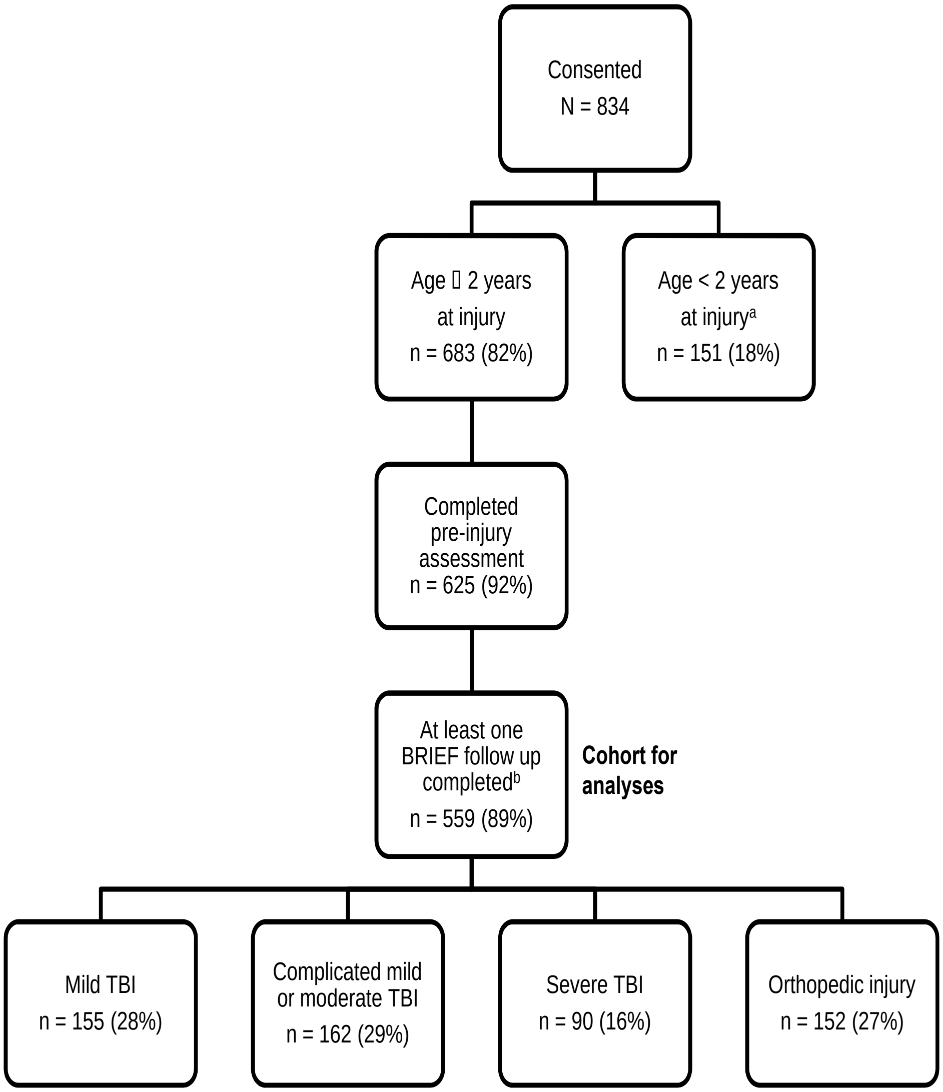

[a] Subjects are not eligible for BRIEF (preschool version) assessments until 2 years of age.
[b] Number of assessments completed at each time point were pre-injury, n=556; 3 months, n=517; 12 months, n=511; 24 months, n=473; and 36 months, n=416.

© 2021 Keenan HT et al. *JAMA Network Open.*

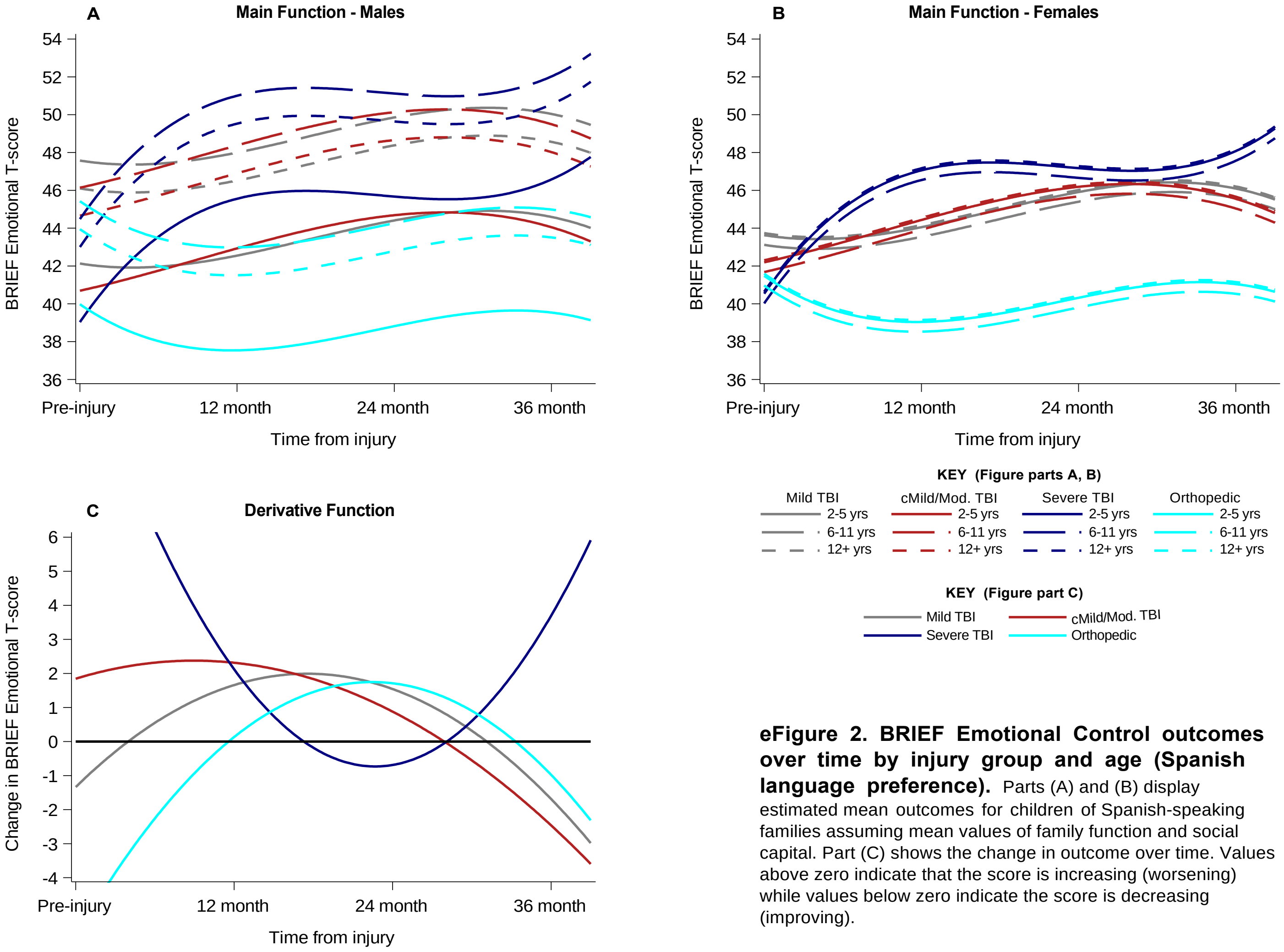

eFigure 2. BRIEF Emotional Control outcomes over time by injury group and age (Spanish language preference). Parts (A) and (B) display estimated mean outcomes for children of Spanish-speaking families assuming mean values of family function and social capital. Part (C) shows the change in outcome over time. Values above zero indicate that the score is increasing (worsening) while values below zero indicate the score is decreasing (improving).

© 2021 Keenan HT et al. *JAMA Network Open.*

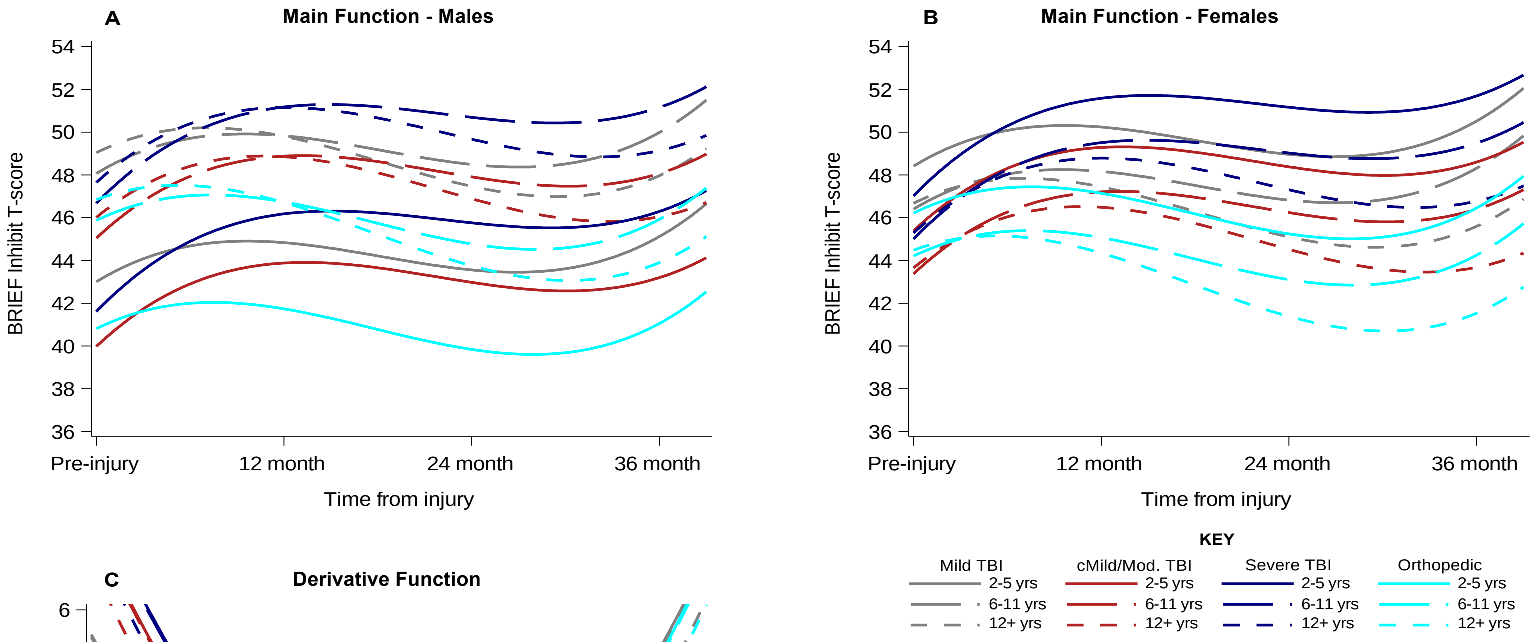

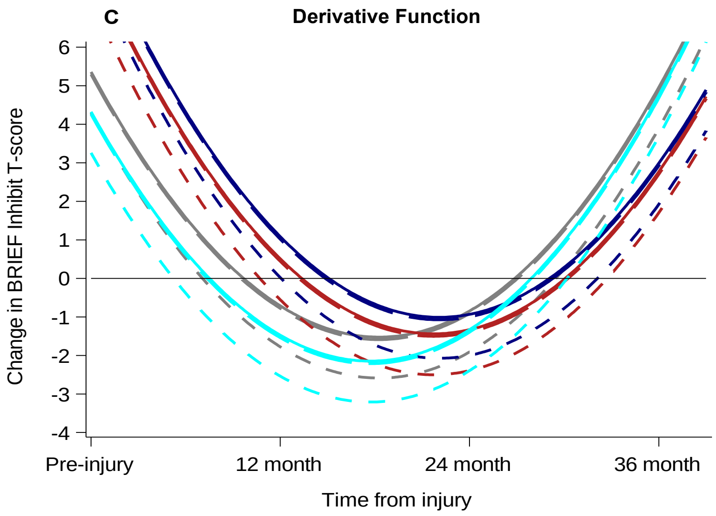

eFigure 3. BRIEF Inhibit outcomes over time by injury group and age (Spanish language preference). Parts (A) and (B) display estimated mean outcomes for children of Spanish-speaking families assuming mean values of family function and social capital. Part (C) shows the change in outcome over time. Values above zero indicate that the score is increasing (worsening) while values below zero indicate the score is decreasing (improving).

© 2021 Keenan HT et al. *JAMA Network Open.*

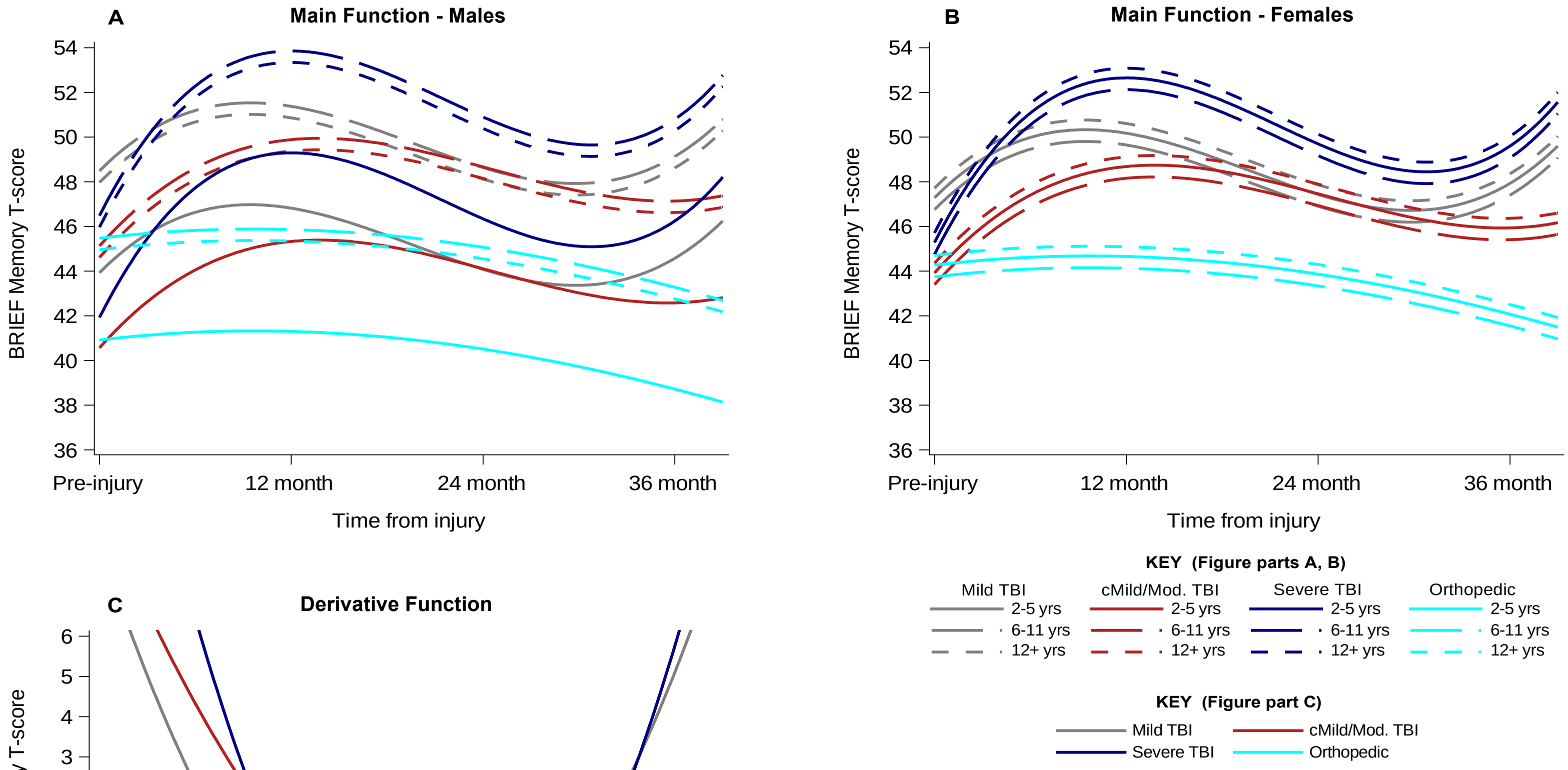

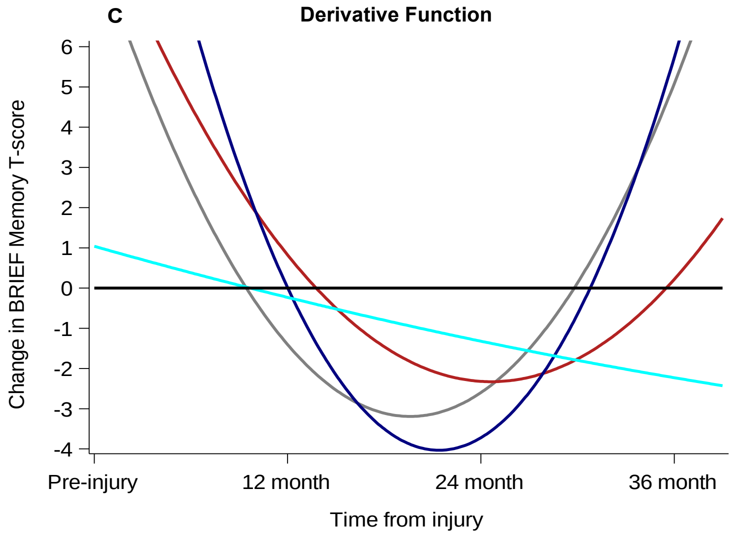

eFigure 4. BRIEF Working Memory outcomes over time by injury group and age (Spanish language preference). Parts (A) and (B) display estimated mean outcomes for children of Spanish-speaking families assuming mean values of family function and social capital. Part (C) shows the change in outcome over time. Values above zero indicate that the score is increasing (worsening) while values below zero indicate the score is decreasing (improving).

© 2021 Keenan HT et al. *JAMA Network Open.*

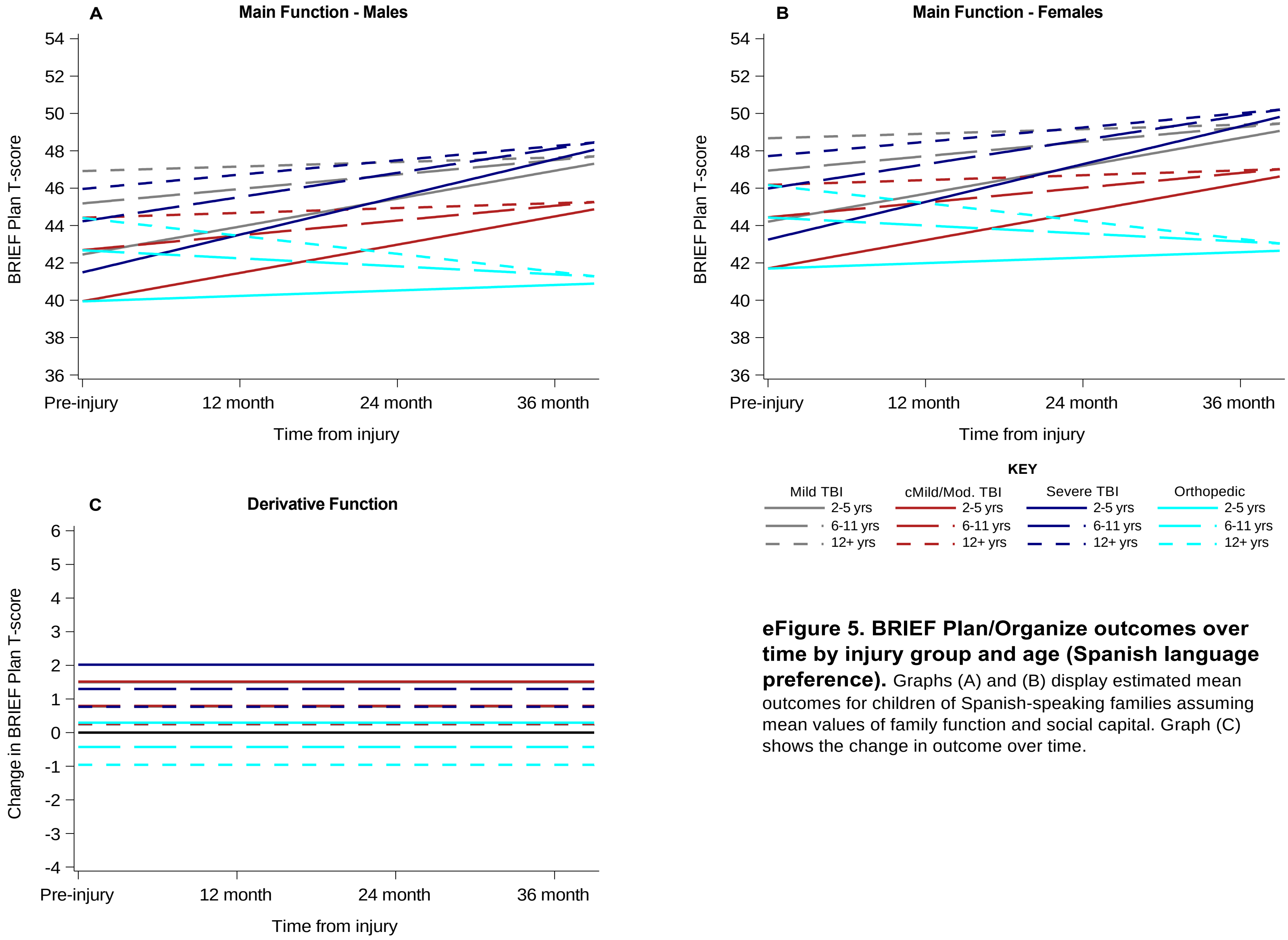

eFigure 5. BRIEF Plan/Organize outcomes over time by injury group and age (Spanish language preference). Graphs (A) and (B) display estimated mean outcomes for children of Spanish-speaking families assuming mean values of family function and social capital. Graph (C) shows the change in outcome over time.

© 2021 Keenan HT et al. *JAMA Network Open.*
